# A Comparative Study of Endoscopic versus Percutaneous Epidural Neuroplasty in Lower Back Pain: Outcomes at Six-Month Follow Up

**DOI:** 10.3390/medicina60050839

**Published:** 2024-05-20

**Authors:** Jong Bum Choi, Jae Chul Koh, Daehyun Jo, Jae Hyung Kim, Won Sok Chang, Kang Taek Lim, Hyung Gon Lee, Ho Sik Moon, Eunsoo Kim, Sun Yeul Lee, Kibeom Park, Yi Hwa Choi, Sang Jun Park, Jinyoung Oh, Sook Young Lee, Bumhee Park, Eun Kyung Jun, Yeong Seung Ko, Ji Su Kim, Eunji Ha, Tae Kwang Kim, Gyu Bin Choi, Ra Yoon Cho, Na Eun Kim

**Affiliations:** 1 Department of Anesthesiology and Pain Medicine, Ajou University School of Medicine, Suwon 16499, Republic of Korea; romeojb@naver.com (J.B.C.); anesylee@aumc.ac.kr (S.Y.L.); tk.kim@aumc.ac.kr (T.K.K.);; 2 Department of Anesthesiology and Pain Medicine, Korea University College of Medicine, Korea University Anam Hospital, Seoul 02841, Republic of Korea; jaykoh@korea.ac.kr; 3 Jodaehyun Pain Center, Jeonju 55149, Republic of Korea; pandjo@naver.com; 4 Department of Anesthesiology and Pain Medicine, Hallym University School of Medicine, Dongtan Sacred Heart Hospital, Hwaseong 18450, Republic of Korea; jaehkim11@gmail.com; 5 Department of Anesthesiology and Pain Medicine, Chungdam Wooridul Spine Hospital, Seoul 06068, Republic of Korea; 6 Department of Neurosurgery, AIN Hospital, Incheon 22148, Republic of Korea; limkat@hanmail.net; 7 Department of Anesthesiology and Pain Medicine, Chonnam National University Medical School, Gwangju 61469, Republic of Korea; leehg@chonnam.ac.kr; 8 Department of Anesthesiology and Pain Medicine, College of Medicine, The Catholic University of Korea, Seoul 06591, Republic of Korea; mhsjshemp@empas.com; 9 Department of Anesthesia and Pain Medicine, School of Medicine, Pusan National University, Busan 50612, Republic of Korea; eunsookim@pusan.ac.kr; 10 Department of Anesthesiology and Pain Medicine, Chungnam National University School of Medicine, Chungnam National University Hospital, Daejeon 35015, Republic of Korea; neoquack@naver.com; 11 Department of Anesthesiology and Pain Medicine, Keimyung University Dongsan Hospital, Daegu 42601, Republic of Korea; pakkibum@naver.com; 12 Department of Anesthesiology and Pain Medicine, Hallym University School of Medicine, Hallym University Sacred Heart Hospital, Anyang 14068, Republic of Korea; pcyhchoi@hallym.or.kr; 13 Department of Anesthesiology and Pain Medicine, Yonsei University School of Medicine, Severance Hospital, Seoul 03722, Republic of Korea; iotas@naver.com; 14 Department of Anesthesiology and Pain Medicine, Kyungpook National University School of Medicine, Kyungpook National University Chilgok Hospital, Daegu 41944, Republic of Korea; sgcms3@gmail.com; 15 Department of Biomedical Informatics, Ajou University School of Medicine, Suwon 16499, Republic of Korea; 16 Office of Biostatistics, Medical Research Collaborating Center, Ajou Research Institute for Innovative Medicine, Ajou University Medical Center, Suwon 16499, Republic of Korea; k.jisu5107@aumc.ac.kr; 17 Department of Anesthesiology and Pain Medicine, Ajou University Graduate School of Medicine, Suwon 16499, Republic of Korea; 18 Department of Anesthesiology and Pain Medicine, Inha University School of Medicine, Incheon 22332, Republic of Korea; col3004@naver.com; 19 Department of Anesthesiology and Pain Medicine, Beomeo First Orthopedic Clinic, Daegu 42087, Republic of Korea

**Keywords:** endoscopic epidural neuroplasty, percutaneous epidural neuroplasty, visual analog scale, Oswestry disability index

## Abstract

*Background and Objectives*: Endoscopic epidural neuroplasty (EEN) facilitates adhesiolysis through direct epiduroscopic visualization, offering more precise neural decompression than that exhibited by percutaneous epidural neuroplasty (PEN). We aimed to compare the effects of EEN and PEN for 6 months after treatment with lower back and radicular pain in patients. *Methods*: This retrospective study compared the visual analog scale (VAS) and Oswestry disability index (ODI) scores in patients with low back and radicular pain who underwent EEN or PEN with a steering catheter. The medical records of 107 patients were analyzed, with 73 and 34 undergoing EEN and PEN, respectively. *Results*: The VAS and ODI scores decreased at all time points after EEN and PEN. VAS and ODI scores decreased more in the EEN group than those in the PEN group at 1 day and 1- and 6-months post-procedure, indicating superior pain relief for both lower back and radicular pain through EEN. *Conclusions*: EEN is a superior treatment of pain control than PEN in lower back and radicular pain patients.

## 1. Introduction

Low back and radicular pain commonly occur in patients with degenerative spondylosis. Low back and radicular pain have many underlying causes, one of which is scarring in the epidural space. Scarring in the epidural space can cause pain for many reasons. The nerves may be trapped by severe adhesion, while congestive veins in the epidural space can become enlarged and exert pressure on the nerves [1].

Percutaneous epidural neuroplasty (PEN) with a wire-type catheter, first reported by Dr. Racz, has been widely practiced since 1989 and reduces pain through epidural adhesiolysis, epidural fibrosis, and inflammation near the neural tissue.

The PEN procedure is used to dissolve the scar tissue around the entrapped nerves in the epidural space of the spine. PEN can be performed percutaneously using a Racz catheter [1]. The catheter may be manipulated to mechanically break up adhesions while various agents such as anesthetics, corticosteroids, hyaluronidase, and hypertonic saline are injected. In endoscopic epidural neuroplasty (EEN), a flexible catheter is inserted into the sacral hiatus to precisely place the injection in the epidural space and onto the nerve root [2,3,4]. Both EEN and PEN can eliminate the adhesion, which can physically prevent the direct delivery of drugs to the nerves and may provide pain relief to patients who have not responded to epidural blocks, physical therapy, or medication [5].

In this study, we compared visual analog scale (VAS) and Oswestry disability index (ODI) scores at 1 day, 1 month, and 6 months after EEN or PEN in patients with low back and radicular pain who had undergone EEN or PEN.

## 2. Materials and Methods

### 2.1. Study Design and Participants

This study was approved by the Ethics Committee of Ajou University Hospital (AJIRB-MED-MDB-20-53) and registered at ClinicalTrials.gov (identifier: NCT05533723) on 9 September 2022. All patients provided written informed consent before undergoing EEN or PEN. Informed consent was not required for this study because only information from the patients’ charts and electronic medical records was used. This study was a retrospective study that included patients with EEN (N = 73) and PEN (N = 34), and through consultation with the statistics department, the results were derived as a fixed effect (VAS) considering age, sex, and time.

This study compared VAS and ODI scores in patients who had undergone EEN or PEN for low back and radicular pain in the Ajou University Hospital Pain Clinic. A total of 107 patients were enrolled, and those who underwent EEN (N = 73) and PEN (N = 34) between 2016 and 2020 were included. The EEN and PEN treatment results were analyzed using data from electronic medical records.

### 2.2. Inclusion and Exclusion Criteria

The inclusion criteria were as follows: (1) age > 20 years; (2) low back or radicular pain in the lower extremities; (3) persistent low back or radicular pain in the lower extremities despite medication and epidural nerve blocks for 6 months; and (4) confirmed spinal stenosis (SS) using lumbar magnetic resonance imaging (MRI).

The exclusion criteria were as follows: (1) systemic infection; (2) skin infection at the injection site; (3) uncontrolled diabetes mellitus; (4) coagulation abnormalities; and (5) a history of allergic reactions to local anesthetics or contrast agents.

Based on these criteria, 107 patients were included, and VAS, ODI, and patient demographic data were collected from electronic medical records. If additional pain occurred in 6 months after the procedure, NSAIDs and physical therapy were combined.

### 2.3. Outcome Assessments and Follow-Up

Based on these criteria, 107 patients were included, and VAS, ODI, and patient demographic data were collected from electronic medical records. If additional pain occurred in 6 months after the procedure, NSAIDs and physical therapy were combined.

In all patients, the VAS score (0 cm = no pain to 10 cm = worst possible pain) was checked before EEN or PEN and at 1 day, 1 month, and 6 months after EEN or PEN from the patients’ electronic medical records.

The ODI questionnaire (10 items, range 0–100; 0 = no disability) values were checked simultaneously, except 1 day after EEN or PEN.

Pain relief factors were analyzed through a single regression analysis in patients who underwent EEN.

### 2.4. Endoscopic Epidural Neuroplasty

**Figure 1:**
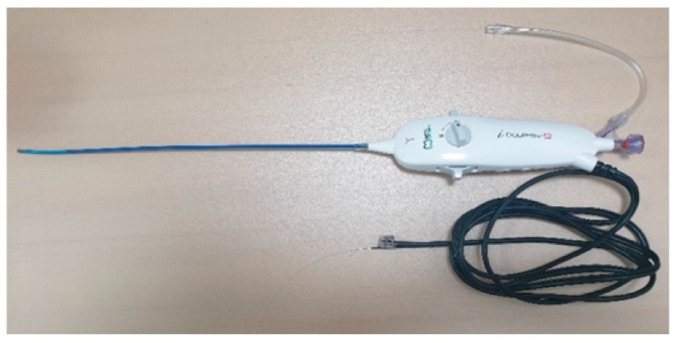
Endoscopic epidural neuroplasty (EEN) catheter, iDolphine S2 (Meta Biomed Co., Ltd., Osong, Republic of Korea). iDolphine S2 is an all-in-one EEN catheter with an epiduroscope, light source, and working channel in one body.

**Figure 2:**
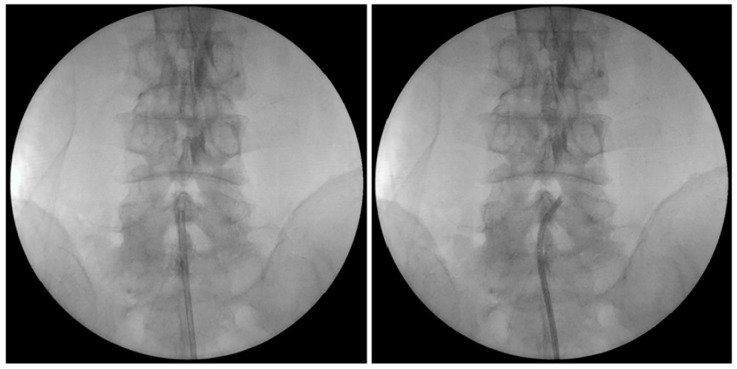
Fluoroscopic view of endoscopic epidural neuroplasty (EEN) with an EEN catheter (iDolphine S2).

**Figure 3:**
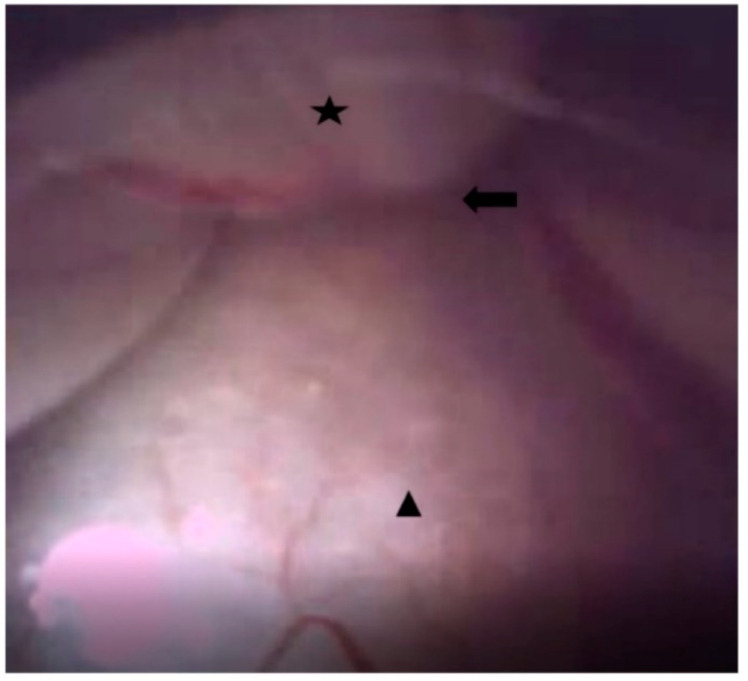
Epiduroscopic view of endoscopic epidural neuroplasty (EEN) with EEN catheter (iDolphine S2); star: anterior wall of the dura mater; triangle: posterior longitudinal ligaments; and arrow: anterior epidural space.

After midnight, nothing by mouth, all patients were placed in a prone position with a pillow, similar to the Wilson frame, under the abdomen after entering the operating room. Blood pressure, pulse rate, and oxygen saturation were measured during EEN in all patients. Under light sedation with hypnotics, and after sterile preparation, a local anesthetic was injected into the sacral hiatus under fluoroscopic guidance, and an EEN catheter, iDolphine S2 (Meta Biomed Co., Ltd., Osong, Republic of Korea), was placed in the epidural space through the sacral hiatus (Figure 1 and Figure 2). EEN was performed on the target segments. During EEN, normal saline was infused into the epidural space under epiduroscopic vision (Figure 3). Dexamethasone (5 mg), 0.3% mepivacaine (10 mL), and hyaluronidase (3000 IU) were injected through the catheter into the target segments. In six patients, the Holmium:YAG laser was used to cut epidural fibrous bands and coagulate epidural bleeding. After EEN, a sterile dressing was applied to the needle entry site. In the recovery room, the patient’s vital signs and side effects, such as motor or sensory abnormalities and post-procedure pain, were closely monitored. When there were no specific abnormalities, the patient was discharged.

### 2.5. PEN with Steering Catheter

**Figure 4:**
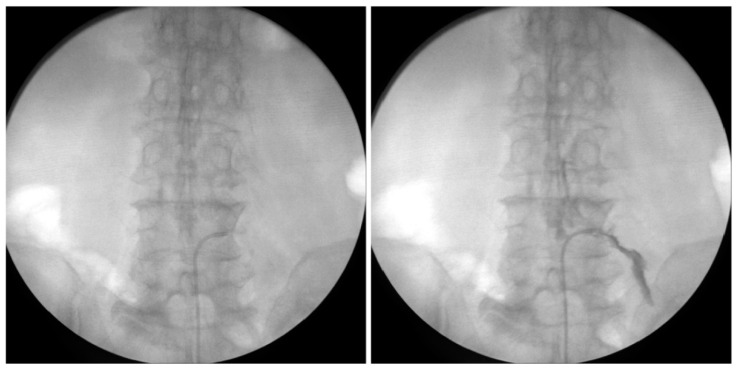
Fluoroscopic view of percutaneous epidural neuroplasty with a steering catheter (EDEN-CC).

**Figure 5:**
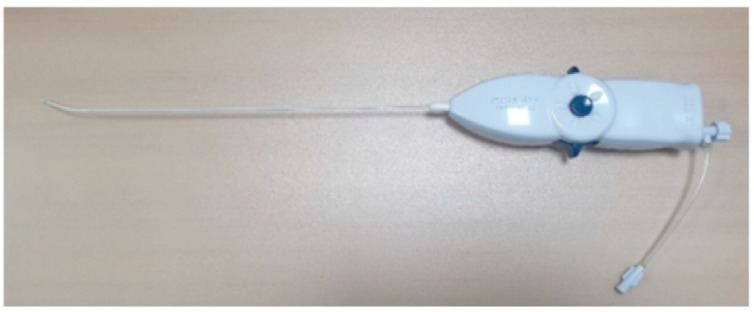
Percutaneous epidural neuroplasty catheter, EDEN-CC (JMT, Yangju, Republic of Korea).

After midnight, nothing by mouth, all patients were placed in a prone position with a pillow under the abdomen. Patients were prepared in the same position using the same method (mentioned above). PEN was carefully performed under fluoroscopic guidance (Figure 4). A PEN catheter, EDEN-CC (JMT, Yangju, Republic of Korea), was placed in the epidural space and neural foramen (Figure 5). PEN was performed on the target segments. Dexamethasone (5 mg), 0.3% mepivacaine (10 mL), and hyaluronidase (3000 IU) were injected through the PEN catheter. After the PEN, a sterile dressing was applied to the needle entry site. In the recovery room, the patient’s vital signs and side effects, such as motor or sensory abnormalities and post-procedure pain, were closely monitored. When there were no specific abnormalities, the patient was discharged.

### 2.6. Statistical Analyses

In this study, the significance level was set at 0.05, with 80% power and a projected dropout rate of 10% used to calculate the sample size. More than 30 patients per group were counted, and a total of 107 patients were included in the EEN (N = 73) and PEN groups N = 34) in this study. All statistical analyses were performed with R software, version 4.2.3. A level of significance of *p* < 0.05 was considered to be statistically significant. A linear mixed effect model was used for comparisons between the two groups and at each time point in each group.

## 3. Results

### 3.1. Participants

The demographic data of the patients are presented in Table 1. This study included 73 and 34 patients who underwent EEN and PEN, respectively (107 patients). There were no statistically significant differences in height, weight, or body mass index (BMI); however, age and procedure time were significantly different (*p* = 0.003 and *p* < 0.001, respectively). There was no change in the dose of the drug, except for gabapentin, during the study period Table 2.

Among the patients who underwent EEN, 17 patients received it on the right side (L4-5:1, L5-S1:13, and L4-5-S1:3), 18 patients received it on the left side (L3-4:1, L4-5:6, L5-S1:7, and L4-5-S1:4), and 38 patients received it bilaterally (L5:8, L4-5:10, L5-S1:16, and L4-5-S1:4).

And the 34 patients who received PEN underwent the procedure as follows: 10 patients received it on the right side (L5-S1:10), 9 patients received it on the left side (L4-5:2, L5-S1:6, and L4-5-S1:1), and 15 patients received it bilaterally (L5:10 and L5-S1:5).

The following medications were taken by patients during the study period in Table 2.

### 3.2. Clinical Outcomes

**Figure 6:**
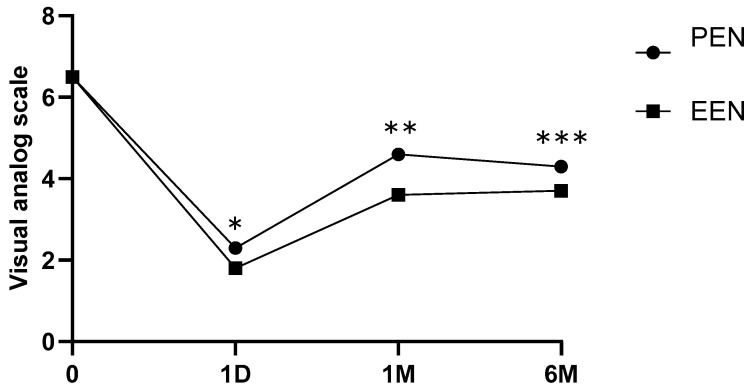
Visual analog scale showing a statistically significant decrease at pre-VAS, 1 day, 1 month, and 6 months after EEN or PEN (EEN: 6.5 ± 0.9, 1.8 ± 0.7, 3.6 ± 0.7, and 3.7 ± 0.8; and PEN: 6.5 ± 0.8, 2.3 ± 0.7, 4.6 ± 1.0, and 4.3 ± 0.7; *, **, *** *p* < 0.05). Mean ± standard deviation of all values is shown for each time point. EEN, endoscopic epidural neuroplasty; PEN, percutaneous epidural neuroplasty.

**Figure 7:**
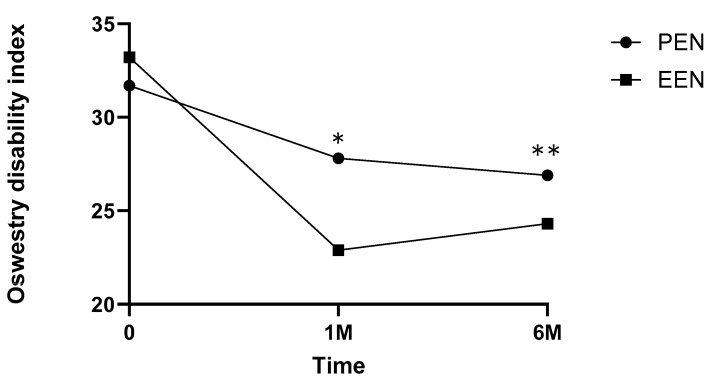
Oswestry disability index scores showing a statistically significant decrease at pre-ODI, 1 and 6 months after EEN or PEN (EEN: 33.2 ± 3.0, 22.9 ± 2.2, and 24.3 ± 3.2; and PEN: 31.7 ± 3.6, 27.8 ± 1.8, and 26.9 ± 2.3; *, ** *p* < 0.05). The mean ± standard deviation of all values is shown for each time point. EEN, endoscopic epidural neuroplasty; PEN, percutaneous epidural neuroplasty.

The VAS scores decreased significantly at 1 day, 1 month, and 6 months after EEN (*p* < 0.001, *p* < 0.001, and *p* < 0.001, respectively) (Table 3, Figure 6). The ODI scores significantly decreased at 1 and 6 months after EEN (*p* < 0.005 and *p* < 0.005, respectively) (Figure 7). For patient size (number of different patients per group), each independent variable was expressed as a percentage, and for repeated comparisons according to age and time, the results obtained through a linear mixed model were as follows (Table 3).

### 3.3. Factors Associated with Pain Relief

A simple regression analysis was performed to correlate the post-procedure VAS and ODI scores with the amount of saline used in EEN, treatment time, and BMI (Table 4).

As the volume of saline used increased, the VAS score decreased significantly at 1 day (*p* = 0.01) and 6 months post-procedure (*p* = 0.03); however, the adjusted R-squared values showed low correlations at 0.1143 and 0.8391, respectively. As the treatment time increased, the VAS score decreased significantly at 6 months post-procedure (*p* = 0.01); however, the adjusted R-squared was 0.1796, indicating a low correlation. There was no association between BMI and postoperative VAS or ODI score (Table 4).

## 4. Discussion

In this study, EEN and PEN were performed, and outcomes were compared in patients with low back and radicular pain.

All patients who underwent EEN and PEN had VAS and ODI reductions for up to 6 months. VAS reductions were greater in the EEN group than those in the PEN group at 1 day and 1 and 6 months after EEN or PEN. ODI reductions were greater in the EEN group than those in the PEN group at 1 and 6 months after EEN or PEN.

At 1 and 6 months after the procedure, EEN was more effective than PEN, as reflected by VAS and ODI scores. The reason for these results was thought to be the powerful and thick EEN catheter; moreover, normal saline infusion via the EEN catheter during EEN may have been effective in irrigating inflammatory materials in the epidural space.

In the ODI analysis, EEN was more effective than PEN at 1 and 6 months after the procedure. These results were thought to be because of various factors that might have influenced the ODI scale, such as physical therapy, exercise, and rehabilitation. The ODI results of this study may have been influenced by long-term care or physical or rehabilitative treatment rather than the VAS score.

Nerve entrapment may result from epidural fibrous tissue remnants after surgery, and the degree of epidural fibrosis can be confirmed using MRI [6]. Severe scarring and adhesions can occur in up to 83–95% of patients after back surgery, and scars that cannot be confirmed on MRI are confirmed in 80% of the cases using epiduroscopy [7]. The authors of this study thought that visualization using epiduroscopy and touching by an endoscopic epidural catheter in EEN were superior to MRI for the diagnosis and treatment of epidural fibrosis and adhesion in PSSS. Epidural fibrosis and scar tissue in this study were confirmed using epiduroscopy in EEN. Adequate pain relief with epidural injections and medications for epidural fibrosis is difficult to achieve. In PSSS, epidural fibrous bands are formed by the posterior mediana dorsalis and anterior Hoffman ligaments. Impaired circulation due to epidural fibrosis in PSSS causes pain, and direct visualization using epiduroscopy can allow effective treatment [8,9,10].

High-volume infusion of normal saline into the epidural space and Holmium:YAG laser treatment of herniated discs in EEN showed efficacy superior to that of PEN. However, in this study, the laser was only used in six of the 73 patients, who did not show a statistically significant pain reduction compared with the EEN group that did not receive laser treatment. However, laser application can cause an inflammatory reaction in the disc and fibrous tissues for up to 4 weeks, which can cause persistent pain. In this study, although the laser was applied only to the fibrous bands in the epidural space, pain reduction persisted for 1 and 6 months after EEN.

A previous study reported a positive correlation between the volume of saline irrigation in the epidural space and radicular or low back pain [11]. In this study, unlike previous studies, there was no correlation between saline infusion volume and post-procedure NRS or ODI. During saline infusion into the epidural space, epidural pressure should be maintained at 50–60 mmHg, and saline should be injected at a rate ≤ 1 mL/s [9]. When performing EEN, it is recommended to use an appropriate amount of fluid (60–250 mL) [3]. In this study, the mean procedure time was 66.84 ± 13.87 min. The mean saline volume used in EEN was 131.31 ± 20.96 mL. The procedure time and volume of normal saline used were appropriate [12]. For scar tissues, such as epidural fibrous tissue, direct visualization and decompression through epiduroscopy are important [13]. A randomized controlled study and a more effective decompression method are needed in the future.

In the EEN group, 3 of the 73 patients underwent dural puncture. After conservative treatment, such as bed rest, hydration, and medication, all symptoms improved. In a previous study, the incidence of dural puncture was 93% when epiduroscopy was performed for several reasons in patients with PSSS [5]. Changes in the anatomical structure due to previous surgery, a decrease in the compliance of the epidural space due to adhesions, and an increase in epidural pressure due to injected saline may cause dural puncture. This can be reduced using equipment with a small diameter or using minimum saline. Dural puncture, visual impairment, intravascular injection, and seizures are major complications that occur in approximately 8% of cases [14]. In this study, no visual impairments, intravascular injections, or seizures were observed in the EEN group. In the PEN group, mild-to-moderate post-procedural pain, but not severe pain, occurred in some patients.

Additionally, it is believed that EEN can be a personalized treatment for lower back pain by quickly controlling pain and returning to daily life through enhanced recovery after surgery [15].

This study has some limitations. First, this study is a retrospective study, so there may be selection bias in the procedures, and it is believed that a randomized controlled study will be needed in the future. Second, previous treatments were not considered for patients with various diseases. Third, the course of more than 1 year was not investigated for both procedures. Since all patients with lower back and radicular pain were targeted without consideration of disease, it is thought that treatment results for each disease will be needed through future large-scale studies.

## 5. Conclusions

All patients who underwent EEN and PEN had VAS and ODI reductions for up to 6 months. VAS reductions were greater in the EEN group than those in the PEN group at 1 day and 1 and 6 months after EEN or PEN. ODI reductions were greater in the EEN group than those in the PEN group at 1 and 6 months after EEN or PEN. EEN can be a better treatment compared with PEN for lower back and radicular pain.

## Figures and Tables

**Table 1:** Demographic data. Among the 73 patients who underwent EEN, 17 patients received it on the right side only, 18 patients received it on the left side, and 38 patients received it bilaterally.

Parameters		EEN (N = 73)	PEN (N = 34)	*p*-Value
Age (Mean, years)		56.20 ± 17.04	67.5 ± 14.04	0.003 *
Sex (M:F)		43:30	19:15	0.304
Height (cm)		163.15 ± 30.66	162.35 ± 8.52	0.887
Weight (kg)		69.72 ± 18.65	65.94 ± 8.50	0.309
BMI (kg/m^2^)		25.19 ± 3.19	25.00 ± 2.56	0.794
Procedure time (min)		66.84 ± 13.87	32.21 ± 6.05	<0.001 *
Diagnosis N (%)	Herniated lumbar disc	43 (0.59)	19 (0.56)	0.772
	Spinal stenosis (mild/moderate/severe, number)	18 (0.25) (0:10:8)	11 (0.32) (0:6:5)	
	Post spinal surgery syndrome	12 (0.16)	4 (0.12)	
Laser used N(%)		6 (0.10)	0	
Used saline (mL)		131.31 ± 20.96	0	

Data are expressed as numbers, means ± standard deviation; * *p* < 0.05; BMI, body mass index; EEN, endoscopic epidural neuroplasty; PEN, percutaneous epidural neuroplasty.

**Table 2:** Medication dose before and after EEN and PEN. There was a statistically significant change in gabapentin dosage during the study period in both groups (*p* = 0.04 and *p* < 0.00).

	**Medication**	**Before EEN**	**6 Months after EEN**	***p* Value**
EEN	Gabapentin dose (mg)	722.6 ± 259.9	651.6 ± 206.1	0.2462
	Pregabalin dose (mg)	275 ± 170.6	260.3 ± 156.9	0.6721
	Tramadol dose (mg)	116.1 ± 48.5	108.4 ± 42.4	0.3119
	Buprenorphine patch dose (µg/h)	7.5 ± 4.5	7.3 ± 3.9	0.8615
	Oxycodon dose (mg)	30 ± 10	25 ± 9.6	0.4382
	Fentanyl patch dose (µg/h)	20.8 ± 5.9	20.8 ± 5.9	1
	**Medication**	**Before PEN**	**6 Months after PEN**	***p* Value**
PEN	Gabapentin dose (mg)	543.8 ± 264.5	475 ± 188.7	0.4197
	Pregabalin dose (mg)	276.4 ± 168	251.4 ± 132.9	0.6337
	Tramadol dose (mg)	120.5 ± 36.6	117.9 ± 37.1	0.7906
	Buprenorphine patch dose (µg/h)	7.5 ± 2.5	5.6 ± 1.7	0.1235
	Oxycodon dose (mg)	20 ± 0	18.3 ± 2.4	0.4227
	Fentanyl patch dose (µg/h)	none	none	none
***p* Value**	**Medication**	**Before PEN**	**6 Months after PEN**	***p* Value**
(EEN vs. PEN)	Gabapentin dose (mg)	0.03984 *	0.0072 *	-
	Pregabalin dose (mg)	0.9771	0.8237	-
	Tramadol dose (mg)	0.6265	0.2821	-
	Buprenorphine patch dose (µg/h)	1	0.1311	-
	Oxycodon dose (mg)	0.0756	0.1949	
	Fentanyl patch dose (µg/h)	none	none	

Data are expressed as dosage of each drugs, means ± standard deviation; * *p* < 0.05.

**Table 3:** Comparison of VAS and ODI scores before EEN or PEN and 1 day, 1 month, and 6 months after EEN or PEN.

	Characteristics	Level	PEN	EEN
VAS	Time	N (%)	34 (0.3)	73 (0.7)
		Pre	6.5 (0.8)	6.5 (0.9)
		immediate_Post	2.3 (0.7)	1.8 (0.7)
		1 mon	4.6 (1.0)	3.6 (0.7)
		6 mon	4.3 (0.7)	3.7 (0.8)
	Age	Mean (SD)	67.5 (14.6)	55.7 (17.4)
	Sex	N (%)	34 (0.3)	73 (0.7)
		M	19 (55.9)	43 (58.9)
		F	15 (44.1)	30 (41.1)
ODI	Time	N (%)	34 (0.3)	73 (0.7)
		Pre	31.7 (3.6)	33.2 (3.0)
		1 mon	27.8 (1.8)	22.9 (2.2)
		6 mon	26.9 (2.3)	24.3 (3.2)
	Age	Mean (SD)	67.5 (14.6)	55.7 (17.4)
	Sex	N (%)	34 (0.3)	73 (0.7)
		M	19 (55.9)	43 (58.9)
		F	15 (44.1)	30 (41.1)

Data are expressed as numbers, means ± standard deviation; *p* < 0.05; VAS, visual analog scale; ODI, Oswestry disability index; EEN, endoscopic epidural neuroplasty; PEN, percutaneous epidural neuroplasty.

**Table 4:** Factors associated with pain relief after EEN.

Independent Variable	Dependent Variable		Beta (95% CI)	*p*-Value
Saline volume	VAS	1 day	−0.003472	0.019 *
		1 month	0.0010754	0.157
		6 months	0.0000911	0.925
	ODI	1 month	0.0007815	0.331
		6 months	0.001533	0.039 *
Procedure time	VAS	1 day	−0.002024	0.222
		1 month	0.0017468	0.075
		6 months	0.002609	0.036 *
	ODI	1 month	0.0000558	0.953
		6 months	0.001148	0.263
Age	VAS	1 day	0.005128	0.003 *
		1 month	0.000629	0.503
		6 months	0.001043	0.371
	ODI	1 month	−0.0004532	0.650
		6 months	0.0002543	0.790
Body mass index	VAS	1 day	−0.0186	0.119
		1 month	−0.002268	0.708
		6 months	−0.009225	0.223
	ODI	1 month	0.006302	0.271
		6 months	0.002679	0.636

* *p* < 0.05; VAS, visual analog scale; ODI, Oswestry disability index; EEN, endoscopic epidural neuroplasty; CI, confidence interval.

## Data Availability

All data used during this study are available upon request from the corresponding author.
